# Upward-looking L-band FMCW radar for snow cover monitoring

**DOI:** 10.1016/j.coldregions.2014.03.006

**Published:** 2014-07

**Authors:** Robert Okorn, Georg Brunnhofer, Thomas Platzer, Achim Heilig, Lino Schmid, Christoph Mitterer, Jürg Schweizer, Olaf Eisen

**Affiliations:** a FH JOANNEUM, Department of Electronic Engineering, Kapfenberg, Austria; b Institute of Environmental Physics IUP, University of Heidelberg, Germany; c WSL Institute for Snow and Avalanche Research SLF, Davos Dorf, Switzerland; d Alfred Wegener Institute Helmholtz Centre for Polar and Marine Research, Bremerhaven, Germany

**Keywords:** Snow stratigraphy, FMCW, Radar, Surface tracking, Snowpack monitoring

## Abstract

Forecasting snow avalanche danger in mountainous regions is of major importance for the protection of infrastructure in avalanche run-out zones. Inexpensive measurement devices capable of measuring snow height and layer properties in avalanche starting zones may help to improve the quality of risk assessment. We present a low-cost L-band frequency modulated continuous wave radar system (FMCW) in upward-looking configuration. To monitor the snowpack evolution, the radar system was deployed in fall and subsequently was covered by snowfalls. During two winter seasons we recorded reflections from the overlying snowpack. The influence of reflection magnitude and phase to the measured frequency spectra, as well as the influence of signal processing were investigated. We present a method to extract the phase of the reflection coefficients from the phase response of the frequency spectra and their integration into the presentation of the measurement data. The phase information significantly improved the detectability of the temporal evolution of the snow surface reflection. We developed an automated and a semi-automated snow surface tracking algorithm. Results were compared with independently measured snow height from a laser snow-depth sensor and results derived from an upward-looking impulse radar system (upGPR). The semi-automated tracking used the phase information and had an accuracy of about 6 to 8 cm for dry-snow conditions, similar to the accuracy of the upGPR, compared to measurements from the laser snow-depth sensor. The percolation of water was observable in the radargrams. Results suggest that the upward-looking FMCW system may be a valuable alternative to conventional snow-depth sensors for locations, where fixed installations above ground are not feasible.

## Highlights

• Snowpack measurement with upward-looking FMCW radar
• Method to extract the phase of the reflection coefficients
• Include phase into the illustration of the measurement data
• Automated and semi-automated snow surface tracking algorithm
• Improved snow surface tracking when using the phase of the reflection coefficients

## 1 Introduction

Continuous upward-looking radar has proven to be a successful technique for monitoring the temporal evolution of snow stratigraphy. This specific radar setup operates from underneath the snowpack at a fixed location and records quasi-continuously snow-related signal reflections over the course of a season (Gubler and Hiller, 1984; Gubler and Weilenmann, 1987; Heilig et al., 2009, 2010; Mitterer et al., 2011a; Schmid et al., 2014). So far, impulse and frequency modulated continuous wave (FMCW) systems have both been applied in various research studies. Gubler and Hiller (1984) were the first to describe the method of upward-looking FMCW radar measurements. In their pioneering work Gubler (1985) and Gubler et al. (1989) showed that the FMCW radar is suitable to determine snow water equivalent, and qualitatively record new snow height, internal layering and total snow height for a dry high-alpine snowpack. Recently, Heilig et al. (2009, 2010) presented feasibility studies on using impulse radar systems (upGPR) from beneath the snowpack with vertically moving antennas to distinguish system related noise from snowpack-related reflection signals. Applying such a system, Mitterer et al. (2011a) showed that it is feasible to determine the snow height during dry-snow conditions and to estimate the bulk volumetric liquid water content (*θ*_W_) as well as to monitor the position of the wet-to-dry transition over an entire season. However, commercially available impulse systems are expensive and the necessary vertical movement of the antennas for improved upGPR signal processing requires significant power supply, either provided via solar panels together with batteries or through a constant power line. Only very few study sites can provide a constant power line and for regions with low solar input (e.g. polar regions during the winter season) battery charge through solar panels is not feasible for extended periods.

Griffiths (1990) and Stove (1992) summarized the theory of FMCW radar. Marshall and Koh (2008) reviewed the theory of FMCW radar with special focus to snow research and gave an overview on previous research applying continuous wave systems in snow. They concluded that the assessment of the optimum frequency range and bandwidth of the FMCW radars depends on expected total snow depths, desired vertical resolution and manufacturing costs. While a large bandwidth in FMCW systems guarantees a high vertical resolution, high frequency ranges are not able to penetrate the snow cover once liquid water is present (Gubler and Hiller, 1984). Furthermore, components for super high frequency ranges are much more costly than those in the cell-phone frequency range of 850 MHz to 1900 MHz.

**Fig. 1:**
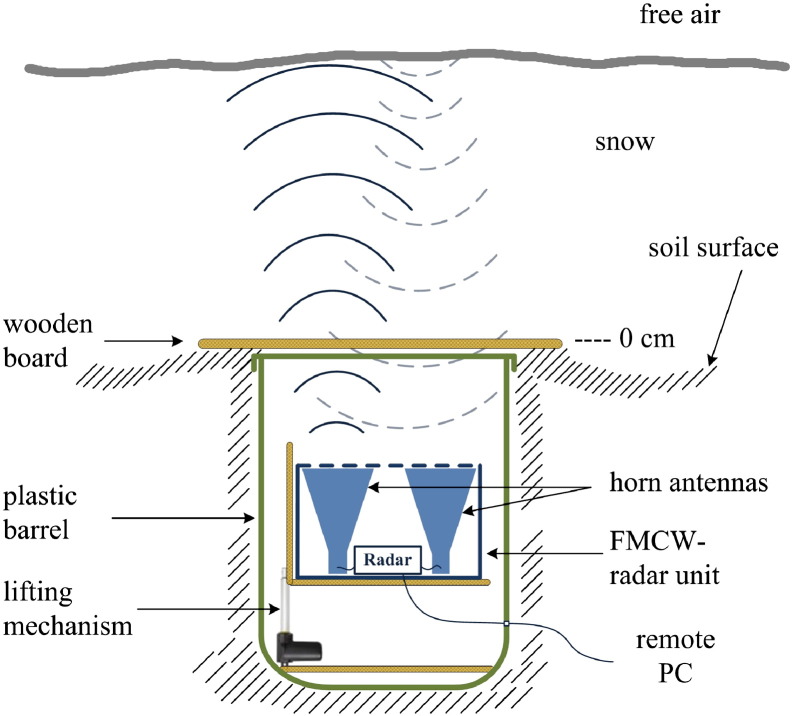
Arrangement of the upward-looking FMCW measurement setup. The radar unit is situated inside a plastic barrel covered by a wooden board providing the zero line. A lifting mechanism enables measurements at different heights.

Despite significant advances in radar applications for snowpack characterization, a low-cost, low-power consuming radar that can be reliably applied in remote locations is presently lacking. We therefore developed a low-cost upward-looking L-band (1–2 GHz) FMCW radar that shall adequately detect the snow surface for alpine snowpacks during the course of a season, even under wet-snow conditions. The system was buried in the ground (Fig. 1). It operated during two winter seasons and recorded reflections from the overlaying snowpack.

## 2 FMCW radar system

### 2.1 Measurement principle

The principle of FMCW radar technology and procedures to increase the performance has been previously described in detail (e.g., Brooker, 2005; Griffiths, 1990; Kurt, 2007; Parthasarathy, 2002; Voges, 1991). In the following we will only describe aspects relevant to our study.

**Fig. 2:**
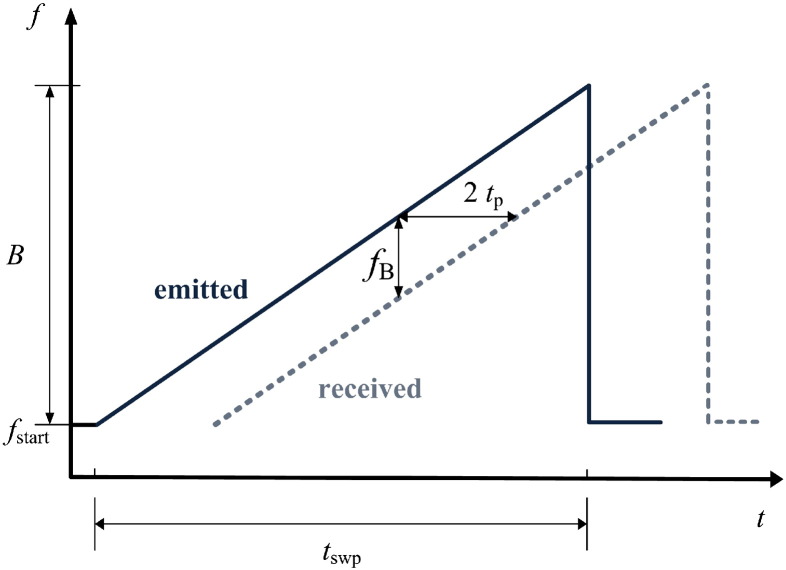
Measurement principle of the FMCW radar system illustrating the relation between emitted and received signals (frequency and time axis are in the order of GHz and ms, respectively). For notation see text.

A radio frequency (RF) signal is emitted that is swept linearly in frequency in a given sweep time *t*_swp_ (Fig. 2). At interfaces of the complex ordinary relative dielectric permittivity ${\underline{\varepsilon }}_{r}=\varepsilon_{r}^{\prime }-j\varepsilon_{r}^{\prime \prime }$ a vertical incident electromagnetic wave is partially reflected according to:

(1) $${\underline{r}}_{i}=|{\underline{r}}_{i}|e^{j\delta_{i}}=\frac{\sqrt{{\underline{\varepsilon }}_{r_{i}}}-\sqrt{{\underline{\varepsilon }}_{r_{i+1}}}}{\sqrt{{\underline{\varepsilon }}_{r_{i}}}+\sqrt{{\underline{\varepsilon }}_{r_{i+1}}}}$$

where $\left|{\underline{r}}_{i}\right|$ is the magnitude of the reflection coefficient and *δ*_*i*_ the reflection phase of the *i*^th^ interface. According to Hallikainen et al. (1986) and Kovacs et al. (1995) the imaginary part (loss factor) of the permittivity of dry snow is in the order of 10^− 3^ in our frequency range and can be neglected. Thus *ε*_r_ and *r*_*i*_ are real-valued and depend only on the dry-snow density. Therefore, the phase of the *i*^th^ reflection coefficient *δ*_1_ approximates either 0° (reflection from interface between more dense and less dense snow or snow to air) or 180° (interface between less dense and more dense snow).

The receiving antenna records delayed and attenuated versions of the transmitted signal, whereat the received signals have propagated twice through the medium between antenna and interfaces. The two-way travel time (*τ* = 2*t*_p_) is related to the reflection distances *D*_*i*_ by

(2) $$\tau_{i}=\frac{2D_{i}}{c}\sqrt{\varepsilon_{r_{{\mathrm{eff}}_{i}}}}$$

where $\varepsilon_{r_{{\mathrm{eff}}_{i}}}$ is the effective dielectric permittivity between the antennas and the *i*^th^ interface, and *c* is the velocity of light in vacuum.

**Fig. 3:**
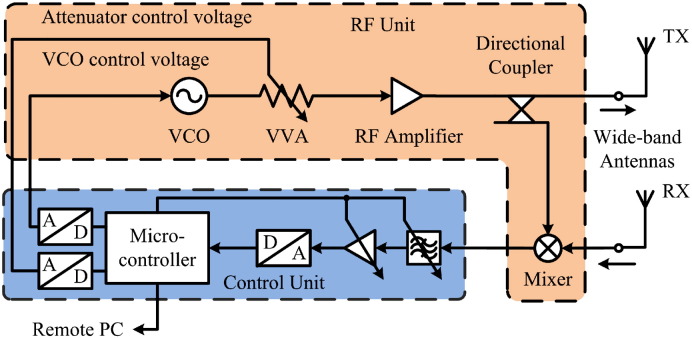
Simplified block schematic of the FMCW radar illustrating the connection between the radar control circuitry and the RF unit.

A double balanced mixer in the receiving path of the radar multiplies the received signals with a portion of the transmitted sweep signal (Fig. 3) and outputs sine wave signals with beat frequencies $f_{B_{i}}$, which lie in the lower kHz range in case of our radar, and frequencies at twice the transmitting frequency. The latter are removed by a subsequent lowpass filter. The obtained beat frequency range can be adjusted by the sweep time *t*_swp_. To achieve beat frequencies in the low kHz range for our application the sweep time has to be in the order of milliseconds. Each beat frequency component $f_{B_{i}}$ is proportional to *τ*_*i*_ by

(3a) $$\tau_{i}=f_{B_{i}}\frac{t_{\mathrm{swp}}}{B}$$

and corresponds to an individual reflection distance

(3b) $$D_{i}=f_{B_{i}}\frac{t_{\mathrm{swp}}}{2B}\frac{c}{\sqrt{\varepsilon_{r_{{\mathrm{eff}}_{i}}}}},$$

provided the distances between adjacent interfaces are greater than the physical radar resolution

(3c) $$|D_{i+1}-D_{i}|>D_{{\mathrm{res}}_{i}}$$

for a given bandwidth *B*.

The FMCW range resolution $D_{{\mathrm{res}}_{i}}$, defined as the minimum distinguishable distance between two adjacent interfaces, has been previously discussed (e.g., Brooker, 2005; Holt et al., 2009; Kurt, 2007; Marshall et al., 2004) and is given by

(4) $$D_{{\mathrm{res}}_{i}}=\frac{c}{2B\sqrt{\varepsilon_{r_{{\mathrm{eff}}_{i}}}}}.$$

For a FMCW radar with a bandwidth of *B* = 1 GHz the range resolution is between approximately 13.8 cm and 10.5 cm in dry snow, assuming snow densities between 100 kg/m^3^ and 500 kg/m^3^, resulting in an effective relative permittivity of $\varepsilon_{r_{\mathrm{eff}}}$= 1.18 to 2.02 (Kovacs et al., 1995), respectively.

Based on the emitted RF signal

(5) $$E\left(t\right)=\hat{E}\sin\left(2\pi f_{\mathrm{start}}t+2\pi \frac{B}{2t_{\mathrm{swp}}}t^{2}+\varphi_{0}\right)$$

and an assumed parallel snow stratification, it can be shown (e.g. Parthasarathy, 2002; Stove, 1992; Voges, 1991) that the beat signal of the *i*^th^ interface on the mixer output in the time domain is independent of the arbitrary initial phase *φ*_0_ and computes to:

(6a) $$u_{B_{i}}\left(t\right)={\hat{A}}_{B_{i}}\cos\left(2\pi f_{B_{i}}t+\varphi \left(f_{B_{i}}\right)\right),$$

where

(6b) $${\hat{A}}_{B_{i}}∼L_{i}\left|{\underline{r}}_{i}\right|\prod_{k=1}^{i-1}\left(1-|{\underline{r}}_{k}|{}^{2}\right),$$

and

(6c) $$\varphi \left(f_{B_{i}}\right)=2\pi \frac{t_{\mathrm{swp}}}{B}f_{\mathrm{start}}f_{B_{i}}-\pi \frac{t_{\mathrm{swp}}}{B}f_{B_{i}}^{2}-\delta_{i}.$$

The amplitude ${\hat{A}}_{B_{i}}$ of the mixer output voltage $u_{B_{i}}\left(t\right)$ is proportional to the magnitude $\left|{\underline{r}}_{i}\right|$ of the *i*^*th*^ reflection coefficient and depends also on the reflection coefficients of the *i* − 1 preceding interfaces. The factor *L*_*i*_ includes all transmission losses. With the sweep time and bandwidth specification given in Table 1 the second term in Eq. (6c) is significantly smaller compared to *δ*_1_ as *B* is in the GHz and $f_{B_{i}}$ is in the kHz range. Therefore the total phase information $\varphi \left(f_{B_{i}}\right)$ of the beat signal comprises two determining terms: a phase shift resulting from the $f_{B_{i}}$ multiplied with the sweep start frequency *f*_start_ and the reflection phase *δ*_1_ of the *i*^th^ interface. Eq. (6c) is reduced to

(7) $$\varphi \left(f_{B_{i}}\right)=2\pi f_{\mathrm{start}}f_{B_{i}}\frac{t_{\mathrm{swp}}}{B}-\delta_{i}.$$

### 2.2 Magnitude and phase determination

We take *N* samples of the beat signal from Eqs. (6a), (6b) and (6c) with sampling frequency *f*_s_ during the sweep period *t*_swp_ = *N*/*f*_s_ and apply a discrete Fourier transform (DFT) with zero padding (e.g. (Lyons, 2010)) of size *mN* (appending (*m* − 1)*N* zeroes, *m* integer). The magnitude and phase of the *k*^th^ spectral line of the positive frequency spectrum (neglecting the negative spectral components) compute to:

(8a) $$|\underline{X}\left(k\right)|=\frac{{\hat{A}}_{B_{i}}}{2}\frac{\sin\left(\mathrm{N\pi}\left(\frac{f_{B_{i}}}{f_{s}}-\frac{k}{\mathrm{mN}}\right)\right)}{\sin\left(\pi \left(\frac{f_{B_{i}}}{f_{s}}-\frac{k}{\mathrm{mN}}\right)\right)}$$

and

(8b) $$\varphi \left(k\right)=\pi \left(\frac{f_{B_{i}}}{f_{s}}-\frac{k}{\mathrm{mN}}\right)\left(N-1\right)+\varphi \left(f_{B_{i}}\right).$$

The spectral line index *k* represents the DFT grid frequencies *f*_grid_(*k*) (i.e. frequency bins) which are integer multiples from the fundamental frequency *f*_s_/*mN*. Replacing $f_{B_{i}}$ in Eq. (3a) by *f*_grid_(*k*) = *kf*_s_/*mN*, the relation between *k* and the two-way travel time *τ*(*k*) obtained from Eq. (3a) (with *f*_s_*t*_swp_/*N* = 1) is

(8c) $$\tau \left(k\right)=\frac{k}{\mathrm{mB}}.$$

For the spectral line index *k*_*i*_ that is closest to the beat frequency (indicated by a peak in the magnitude spectrum)

(9) $$k_{i}≅\frac{f_{B_{i}}}{f_{s}}\mathrm{mN},$$

the first term in Eq. (8b) is approximately zero. Therefore $\varphi \left(k_{i}\right)≅\varphi \left(f_{B_{i}}\right)$ with a maximum phase error of *π*/2*m* (if the beat frequency is between two adjacent frequency bins). This allows extracting the phase *δ*_1_ of the reflection coefficient by correcting the phase *φ*(*k*_*i*_) by the known radar parameters and the detected beat frequency.

**Fig. 4:**
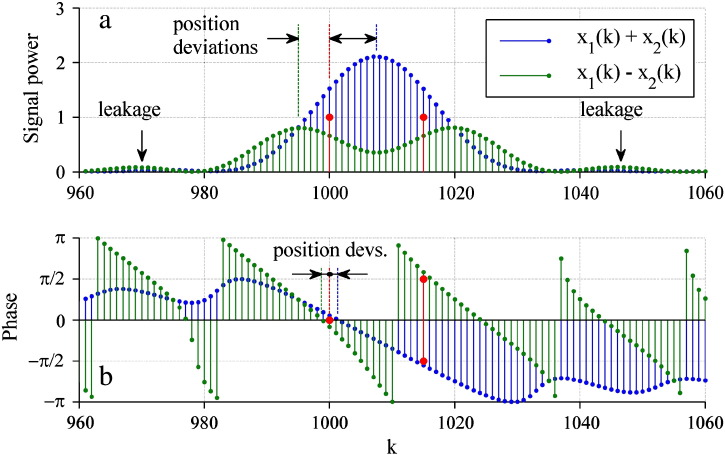
(a) Signal power and (b) phase from two layer interfaces separated by a distance of 0.75 *D*_res_. Red points show true peak positions with expected magnitudes and phase values for additive (*δ*_1_ = *δ*_2_ = 0° = 0°) and subtractive (*δ*_1_ = 0°, *δ*_2_ = 180°) superposition.

In practice, there are multiple reflections resulting in overlaying spectra and with additional peaks from spectral leakage — a side effect of a discrete Fourier transform due to a finite sample count *N* (e.g., Lyons, 2010). This makes the detection of exact beat frequencies difficult. For getting correct peak locations (i.e. correct beat frequencies) in the magnitude spectrum, we found by computer simulations that the minimum distance of adjacent layers had to be approximately 1.5 *D*_res_. If this condition was violated the resulting magnitude spectrum was not accurate. Depending on the individual reflection phases, and due to spectral leakage, the two peaks appeared at deviating positions or superimposed to one peak (Fig. 4 a). However, in the phase spectra the phase values at the true peak locations, and thereby the extracted reflection phases, were close to their expected values (Fig. 4 b). Therefore the phase spectra can indicate the exact positions of the beat frequencies (i.e. interfaces) with fewer deviations than the magnitude spectra.

### 2.3 Measurement setup

We installed a FMCW radar system during the winter seasons 2010–2011 and 2011–2012 at the test site Weissfluhjoch (2540 m a.s.l.) above Davos, Switzerland. In addition to the radar setup, various sensors recording meteorological and snow cover properties were available at this flat test-site during the measurement campaign (Marty and Meister, 2012; Mitterer et al., 2011a). An upward-looking impulse radar system was located in a distance of about 3 m from the upFMCW (Mitterer et al., 2011a; Schmid et al., 2014).

The radar unit consisting of the radar control circuitry, the RF unit, the battery and two broad band horn antennas were sealed inside a plastic box (Fig. 1). The entire FMCW radar system was mounted inside a plastic barrel for protection against meltwater. For the winter 2011–2012 a lifting mechanism was additionally installed that provided two different positions for radar recordings (Fig. 1). In the upper position the antennas were located 14 cm, in the lower position 33 cm below the soil surface. An impregnated wooden board, leveled with the soil surface, covered the pit in which the barrel with the radar was placed. The wooden board protected the radar against the snow load, but more importantly produced a strong partial reflection which provided the zero level in our radargrams. Every 3 h a radar measurement was performed during dry-snow conditions. In spring, to account for fast changes caused by meltwater, upFMCW measurements were conducted every 30 min during the day and again reduced to 3 h intervals during night. Retrospectively, in terms of snowfall detection a 1 h interval seems most appropriate. The raw data were transferred to a remote personal computer where they were processed, visualized and archived. A summary with the operating specifications of the upFMCW radar is given in Table 1. An approximate link budget is provided in Appendix A.

## 3 Data processing

### 3.1 Raw data processing

Initially, we used an average subtraction to DC filter the sampled beat signals within the time domain. In order to avoid unwanted spectral components produced by spectral leakage, time domain windows were applied on the filtered radar data. Similar to Gubler and Hiller (1984), we chose a Kaiser–Bessel window for this processing step. However, the method to track the snow surface presented below yielded better results in terms of tracking precision without using windows (although with the drawback of spectral leakage). In a second step, a discrete Fourier transform was performed using the filtered and windowed signal. We used zero-padding with *m* = 20 to increase the accuracy of absolute range measurement and to decrease the maximum phase error of *π*/2*m*. For echoes that did not violate Eq. (5), the range measurement inaccuracy could be decreased to below 1 cm, the maximum phase error to below 4.5°. The output of the Fourier transform was power and phase spectra, containing the reflected power and phase information of the detected interfaces (from Eqs. (8a), (8b), (8c) and (10)). Constant instrument related influences (static offsets) to power and phase were removed by instrument calibration.

The periodic measurements during the whole winter season resulted in a time series of frequency spectra. All single power and phase spectra of a season were merged together into one radargram in which signal reflections were displayed as a combination of signal power and phase sequences. This presentation allows following the temporal evolution of layers at the surface or within the snowpack and displays the phase characteristics at the surface over the whole season.

### 3.2 Reflection phase estimation

To obtain the phase information *δ*_1_ of the reflection coefficients we had to extract them from the phase spectra *φ*(*k*) by subtracting the constant system phase shift (from beat signal preconditioning) and the $f_{B_{i}}$ related phase shifts as presented in Eqs. (7) and (8b). Due to the fact that multiple reflections may produce overlaying spectra, the number of reflection layers and their exact beat frequencies are a priori unknown. We therefore treated every frequency bin of the phase spectra as a possible beat frequency $\left(f_{B_{i}}=f_{\mathrm{grid}}\left(k\right)\right)$ instead of trying to detect all individual beat frequencies for the $f_{B_{i}}$ related correction. With this approach the first term in Eq. (8b) was zero for every *k*. We substituted $\varphi \left(f_{B_{i}}\right)$ in Eq. (8b) by Eq. (7), replaced $f_{B_{i}}$ by *f*_grid_(*k*) and the latter by *f*_grid_(*k*) = *k f*_s_/*mN*. After rearrangement of Eq. (8b) the extracted phase ${\tilde{\delta }}_{k}$ can be given as:

(10) $${\tilde{\delta }}_{k}=2\pi f_{\mathrm{start}}\frac{f_{s}}{\mathrm{mN}}\frac{t_{\mathrm{swp}}}{B}k-\varphi \left(k\right)-\varphi_{\mathrm{system}\left(k\right)}.$$

Applying this method, we obtained approximately correct phase information $\delta_{i}≅{\tilde{\delta }}_{k}$ at the true beat frequencies. To include the reflection phase information into the radargrams we converted the phase ${\tilde{\delta }}_{k}$ into a sign information. We subtracted *π*/2 from the absolute value of ${\tilde{\delta }}_{k}$ and multiplied the sign of these results with their corresponding reflection power. Accordingly, a peak in the power spectrum with a positive sign represents a reflection phase with *δ*_1_ = 180°, whereas a negative peak represents a phase of *δ*_1_ = 0°.

**Fig. 5:**
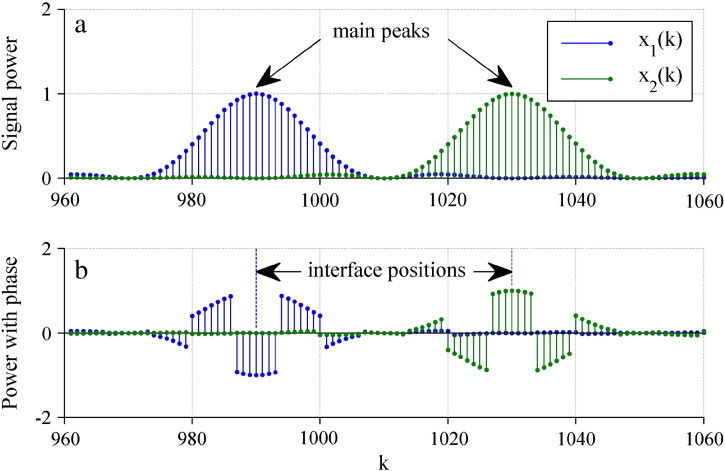
(a) Power spectrum and (b) power multiplied with sign of $|{\tilde{\delta }}_{k}|-\pi /2$ for two different layer interfaces. First interface (blue) with *δ*_1_ = 0°, second interface (green) with *δ*_2_ = 180°.

Due to the fact that not every frequency bin is a true beat frequency, Eq. (10) delivered phase offsets of approximately ± Δ*kπ*/*m* outside of the true beat frequency bins (∆*k* = offset to a true beat frequency bin). That is why the method divided the main peak of an individual reflection in the power spectrum into a sequence of sign changes (Fig. 5). Reflections from interfaces between less dense and more dense snow layers (*δ*_1_ = 180°) are therefore shown as a sequence of 3 positive discontinued by 2 negative magnitude cycles. The reflection position can be defined as the maximum magnitude of the center cycle, which should coincide with the peak of the power spectrum. Sequences with reversed magnitude cycles indicate interfaces between more dense and less dense snow layers or snow to air transitions (*δ*_1_ = 0°). In addition, all radargrams with phase information were processed with median filters to eliminate constant reflections occurring from multiples of the wooden board or from the surrounding bedrock. We applied a 6-weeks moving-window consistency filter. The median of the samples at constant range over 6 weeks (time direction) was subtracted for every trace. In range direction, an additional median filter over 6 samples had to be applied to remove artifacts and uncertainties of the phase conversion.

### 3.3 Snow surface tracking algorithms

**Fig. 6:**
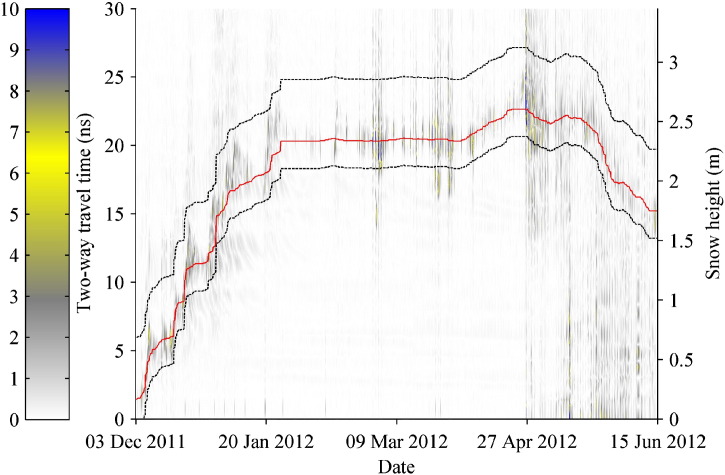
Difference spectrum from winter 2011–12 with smoothed surface trend (red) and limits (black) for peak detection. An arbitrary scale in the range 0 to 10, represented by colors, is used to show the magnitudes of differences.

The snow surface is exposed to changing environmental conditions, such as snow precipitation, snow transport by wind or solar radiation. Thus, the properties of near-surface layers change more readily than those in the lower snowpack, which mainly undergo settlement and metamorphism. Therefore, we developed a simple algorithm (fullyAuto) to automatically track the snow surface which is based on magnitude changes between the successive 3 and 1/2 h measurements. In the first step, the algorithm subtracted the magnitude spectra (magnitude differences between spectral lines of same frequency bin) of consecutive measurements and calculated the absolute values of these differences (referred to as difference spectrum), resulting in a time series of difference spectra. For spike reduction, a moving average filter over 5 samples in time-direction (5 consecutive difference spectra) and 5 samples in range-direction (5 frequency bins of the same trace) was applied, resulting in a time series of smoothed difference spectra (Fig. 6). Then the locations of the maxima in the smoothed difference spectra were determined, which provided an initial trend of the snow surface. In the second step, a median filter was applied to the location vector of the snow surface (i.e. the time series of snow height) to obtain a surface trend (red line in Fig. 6). Constant offsets of + 4.5 ns and − 2 ns (estimates from first snowfall and first settling in the winter season) were added to get the boundaries (black lines in Fig. 6); the surface reflections had to be within. In the third step, the maxima search from the first step was repeated between these boundaries. In the last step the algorithm calculated the positions of the maximum magnitudes in the magnitude spectra with the closest distance to their corresponding maxima in the smoothed difference spectra. These positions were considered as snow surface.

For the semi-automated surface pick (semiAuto), we used the phase information. As mentioned in Section 3.2 the maximum magnitude of a center cycle with negative phase is considered to be a reflection position with *δ*_1_ = 0° (i.e. surface reflection) and should always appear as strongest negative amplitude. This semi-automated pick requires manual interaction as described in Schmid et al. (2014). We used the function “phase follower” of the software package REFLEXW (Sandmeier, 2011), which always follows the peak of the same phase. In case the phase sequence changed or the followed phase disappeared, we checked whether the height of the snow surface changed due to precipitation or melt. If phase variations occurred without new snow or melt events, we ranked consistency in the followed phase higher than picking always the maximum magnitude of a negative phase. During accumulation and melt events, manual intervention was required to reset the follower to the correct phase.

## 4 Results and discussion

Fig. 9: (a) Snow height measured in the winter season 2011–2012 with a laser snow-depth sensor (red), determined with the upward-looking FMCW radar system (lower antenna position) using the fully automated snow surface tracking algorithm fullyAuto (black dots), the semi-automated algorithm semiAuto (green) and determined with an upGPR system using the semi-automated algorithm proposed by Schmid et al. (submitted) (blue). Red circles indicate snow heights measured manually directly above the radar. (b) Differences between the radar determined snow heights and measurements made with the laser snowdepth sensor. The blue background shows when the snowpack was fully wet (according to lysimeter measurements nearby).

**Fig. 7:**
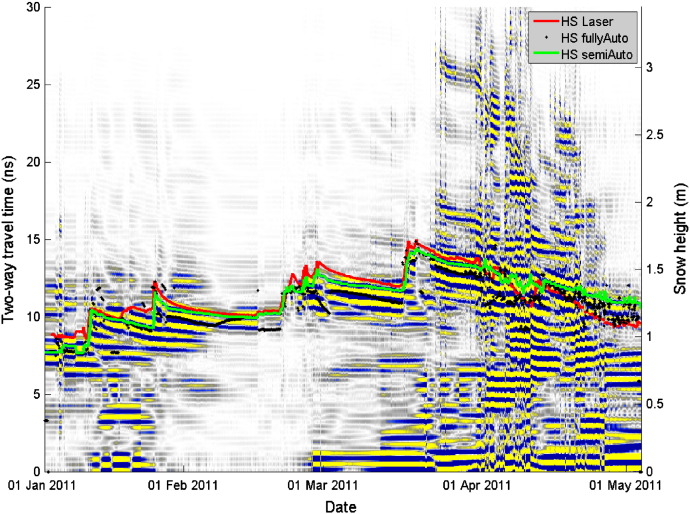
Radargram for the winter 2010–2011 season recorded at Weissfluhjoch. Blue color represents large negative (*δ*_1_ = 0°) and yellow large positive (*δ*_1_ = 180°) magnitudes of reflected signal power multiplied with $\mathrm{sign}\left(|{\tilde{\delta }}_{k}|-\pi /2\right)$. Black diamonds show snow height determined with the fullyAuto snow surface tracking algorithm, the green line indicates the results obtained with the semiAuto algorithm; the red line represents the snow height measured with a laser gauge. A constant wave speed *v* = 0.23 m/ns was assumed to convert two-way travel time into a length scale.

**Fig. 8:**
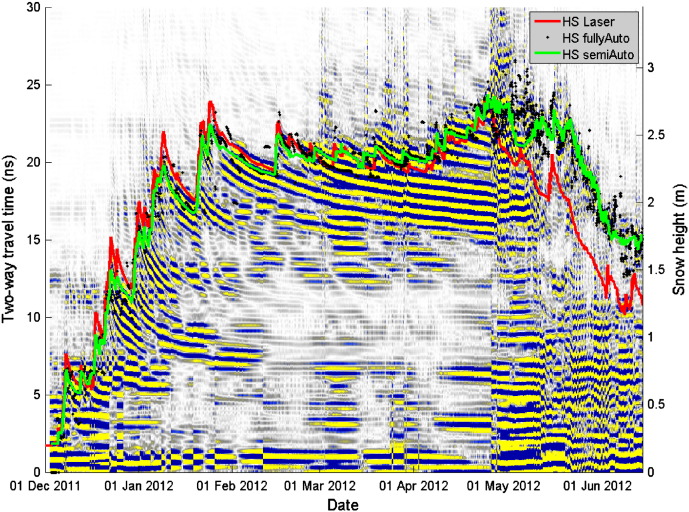
Radargram for the winter 2011–2012 season recorded at Weissfluhjoch (lower antenna position). Same representation as in Fig. 7.

Figs. 7 and 8 show radargrams recorded with the upFMCW radar for the winter seasons 2010–2011 and 2011–2012, respectively. The processed radargrams include already the phase information (Section 3.1 and Eq. (10)). Highly saturated colors indicate large magnitudes, i.e. strong changes in permittivity. According to the definition in Section 3.2, the position of layer transitions was defined as the maximum magnitude of the central phase cycle (Fig. 5).

### 4.1 Snow height determination

Snow height determined with the fully automated picking algorithm (fullyAuto) and the semi-automated algorithm (semiAuto) as well as measured with a laser snow-depth sensor have been plotted into the radargrams (Figs. 7 and 8). In order to compare the snow height determined with the radar with values from the laser, we assumed a constant wave speed of *v* = 0.23 m/ns (Mitterer et al., 2011a) which for dry snow corresponds to a bulk density of 360 kg/m^3^ (Kovacs et al., 1995).

**Fig. 9:**
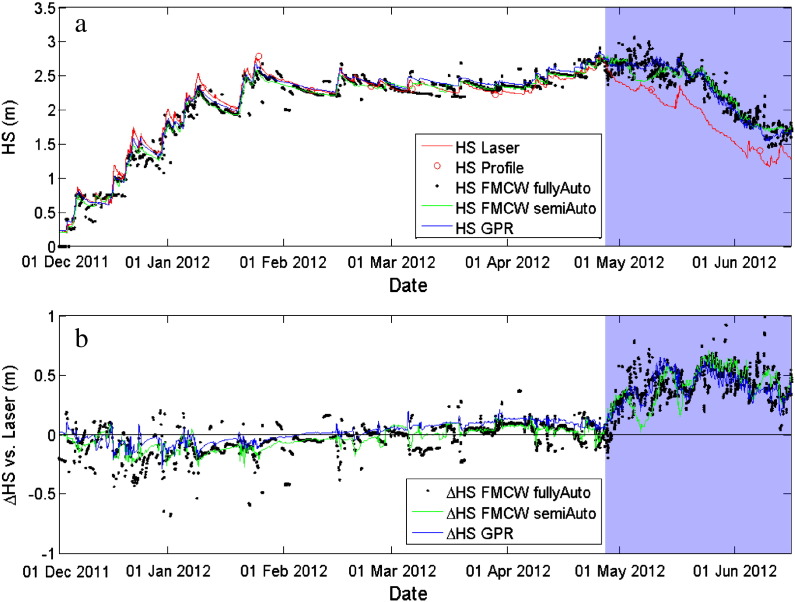
(a) Snow height measured in the winter season 2011–2012 with a laser snow-depth sensor (red), determined with the upward-looking FMCW radar system (lower antenna position) using the fully automated snow surface tracking algorithm fullyAuto (black dots), the semi-automated algorithm semiAuto (green) and determined with an upGPR system using the semi-automated algorithm proposed by (Schmid et al., 2014) (blue). Red circles indicate snow heights measured manually directly above the radar. (b) Differences between the radar determined snow heights and measurements made with the laser snow-depth sensor. The blue background shows when the snowpack was fully wet (according to lysimeter measurements nearby).

The snow height determined with the radar was in general in good agreement with the measurements from the laser snow-depth sensor (Figs. 7, 8 and 9, Table 2). Figs. 7 and 8 show that in particular during dry-snow periods the phase sequence of the surface reflection was stable. The surface height measured by the laser snow-depth sensor was parallel to a curve with constant phase and thereby parallel to the picked semiAuto value, which consistently followed the center of the second negative half-cycle of the surface signal (Section 3.2). This suggests that the phase retrieval algorithm (Eq. (10)) produced correct results and that the retrieved phase information clearly shows height and trend of the snow surface, but also of strong reflecting interfaces within the snowpack. In contrast the magnitude peaks from the automatic surface tracking, which is not using the phase information, fluctuated considerably and thus coincided only occasionally with the measured snow height.

For the entire dry-snow season, the deviation of the fullyAuto routine is larger than of the semiAuto algorithm indicating that the accuracy of snow surface tracking improved when phase information was included. Maximum differences for the fullyAuto reached values up to ± 0.4 m (Fig. 9b). The fullyAuto algorithm had in particular problems when snow layers close to the snow surface such as melt-freeze crusts produced large reflections (15 February–12 March in Fig. 8). Although the surface signal was clearly visible, the algorithm tended to track the larger echo from below the surface. The same occurred for periods of snowfall: the fullyAuto underestimated in most cases the new-snow height. Only for minor snowfalls, the algorithm seemed to work well (e.g. 19 March 2012). The semiAuto algorithm had similar problems in detecting the correct height during snow storms, but performed well in following the reflection of the snow surface during periods of snow settling and low or no accumulation.

Comparing the snow heights from the FMCW radar in the upper and the lower position, no significant differences were observed (Table 2). In addition, post processing the different recording heights by merging the radargrams from both positions with a subsequent stack did not significantly improve noise reduction (not shown here). Thus, the initial idea of a better processing by eliminating system related constant signal noise through the antenna movement was not supported. Contrary to upGPR recordings (Schmid et al., 2014), a lifting mechanism was not necessary for reliable analysis and did not provide further insights. The advantage of the upFMCW radar over the upGPR setup is the fact that power consumption is reduced and the possibility of a mechanical failure of the hoisting system is eliminated.

We concurrently performed radar recordings with a 1.6 GHz upGPR antenna situated 3 m apart from the upFMCW. Fig. 9 shows the snow height extracted from the upGPR signal using the semi-automated tracking approach used by Schmid et al. (2014). Snow heights retrieved from the upGPR signal fitted the laser-determined snow height somewhat better than the upFMCW algorithms for the first part of the season. After 1 March 2012, snow heights retrieved from upFMCW algorithms were closer to the measurements of the laser snow-depth sensor than the ones determined with the upGPR. Considering the entire dry-snow season, the snow heights retrieved from upFMCW algorithms represented the measured snow height similarly accurate as the upGPR (Table 2). Unless at the very beginning of the season, both systems performed very well: snow heights determined with the upward-looking radars were within or less than 10% deviation of the measured snow height during dry-snow conditions. Differences of snow height between upGPR and upFMCW were almost parallel over the entire season suggesting that the offset may have been due to lateral variations in snow height.

The upFMCW tracking algorithms mostly underestimated the snow height at the end of snow storms by 20–25 cm (January 2012 in Fig. 9b), but also with the upGPR some deviations for snowfall events existed. In both cases, they are attributed to the fact that we assumed a constant bulk density of 360 kg/m^3^. Consequently, we overestimated the density and thus underestimated the wave propagation velocity in layers with low density. Nevertheless, the upGPR seemed to slightly better depict the correct snow height during snow storms (Fig. 9a), which probably follows from the higher vertical resolution due to the larger bandwidth (~ 1.6 GHz) of the upGPR in comparison to the upFMCW (1 GHz).

### 4.2 Liquid water within the snowpack

**Fig. 10:**
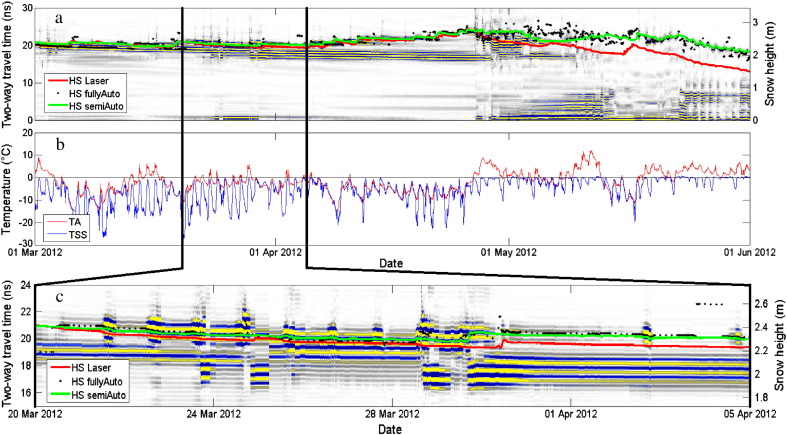
(a) Radargram for the period when the snowpack became wet for the season 2011–2012 (lower antenna position), with decreased color saturation. Same representation as in Fig. 7. (b) Air (red) and snow surface temperature (blue) for the same period. (c) Detail showing a period with melt-freeze cycles (daily from 21 to 29 March) and water percolation down to a snow height of 2 m at 23–24 March and 28–29 March 2012.

The bulk permittivity of the snowpack highly depends on the liquid water content (*θ*_W_). Consequently, presence of water alters the position and magnitude of reflections. In addition, assuming a constant wave velocity is no longer valid since water significantly decreases the wave speed. As a consequence, snow height was overestimated as soon as considerable parts of the snowpack became wet (Fig. 9). Similar to Gubler (1997), we could qualitatively identify periods of wetting and refreezing. Decreasing the color saturation in Fig. 8 allowed us to detect periods and parts of the snow cover with presence of liquid water (Fig. 10a). A first distinct warming at the beginning of March wetted the snow surface, so that the surface temperature occasionally reached 0 °C (Fig. 10b). A distinct cooling followed this first warm period and consequently the water refroze forming a melt-freeze crust. From late March until the beginning of April several such cycles produced water at the snow surface (Fig. 10c). It appears that the meltwater percolated down to a snow height of 2 m or 17.5 ns (~ 0.3 m beneath the surface) and subsequently formed a thick melt-freeze crust within the snowpack. The next period with high air temperatures (last week of April) again, produced considerable water at the snow surface, which first was blocked by the thick melt-freeze crust and probably drained to the bottom of the snowpack within the first days of May 2012. It was, however, very difficult to determine the exact date because the processing to identify and calibrate the record for the reflection originating from the board covering the upFMCW was difficult for the period around 1 May.

Periods when the surface signal reflection disappeared or was hardly detectable are indicated by large scattering of the surface when picked with the fullyAuto algorithm (Fig. 10a, 1–15 May 2012). The semiAuto algorithm followed a constant phase even though the amplitudes were very low. It is very likely that both picks did not always match the snow surface. This period of uncertain surface picks was about two weeks long and was characterized by high air temperatures (Fig. 10b) leading to high *θ*_W_ values, followed by a subsequent snowfall of ~ 20 cm. These circumstances led to very challenging snowpack conditions for both radar systems.

For wet-snow conditions, we numerically determined minimum RMS errors for varying wave speeds ranging from 0.17 to 0.24 m/ns for the semiAuto picks.

Different mean wave speeds and RMS errors were calculated for the seasons 2010–2011 and 2011–2012. For the season 2010–2011, the error was 9 cm for a mean wave speed of *v* = 0.22 m/ns and for 2011–2012 the minimum error was 19 cm for *v* = 0.191 m/ns. In 2011–2012, we recorded upFMCW data for more than 5 weeks longer (until 15 June 2012) than in 2010–2011 when measurements stopped in early May (Figs. 7, 8). However, the mean wet-snow wave speeds for both years will lead to errors of 12.5 cm (2010–2011) and 23.3 cm (2011–2012). It remains debatable, whether a mean wet-snow wave speed is valid for other test sites and whether RMS errors stay comparably low over a full wet-snow season.

Even though considerable amount of water was present within the snowpack during the melt period, it was almost always possible to detect the snow surface with the upFMCW. The results showed that it is generally feasible to detect water within the snow. This offers the possibility to track the position of liquid water, which is thought to be crucial in determining the onset of wet-snow avalanche activity (Mitterer et al., 2011b).

## 5 Conclusions

We developed a low-cost upward-looking FMCW radar, which allowed for continuous monitoring of the snow cover. Since most parts of the custom-made radar system were components used in the production of cell phones, they were highly available and considerably less expensive than those used for e.g. GPR systems. The developed L-band radar was able to capture the temporal evolution of snow height and presence of water within the snowpack during two winter seasons at the test site Weissfluhjoch above Davos, Switzerland. We introduced two tracking algorithms to determine the snow height. The fully automated algorithm (fullyAuto) used only magnitude information from consecutive reflections, while the semi-automated algorithm (semiAuto) used a combination of reflection magnitude and phase information. Results suggest that including the phase information is crucial to improve accuracy for snow height determination. When compared to the snow height measured with a conventional sensor and the snow height determined with an upward-looking GPR, deviations were about 10% or less during the dry-snow seasons. During snowfall both tracking algorithms underestimated snow height due to the fact that a constant bulk density of 360 kg/m^3^ has been assumed. The presence and evolution of water could be captured qualitatively with the upFMCW.

The presented sensor has a robust design and allows many applications. It might be applied for improved data coverage in notoriously difficult-to-forecast avalanche paths or in regions with permanent snow covers, such as the accumulation zones of ice masses like glaciers and ice sheets.

## Figures and Tables

**Table 1:** L-band upFMCW radar specifications.

Frequency range (GHz)	1–2
Bandwidth (GHz)	1
Pulse length (ms)	10
Pulse repetition frequency (Hz)	50
Transmit power (mW)	100
Sampling frequency (kHz)	51.2
Receiver sensitivity (dBm)	− 60
Operating power consumption (W)	1.2
Radar dimensions (width × depth × height) (cm^3^)	60 × 30 × 41
Radar mass (kg)	8.5

**Table 2:** RMSE of two different snow surface tracking algorithms for the upFMCW system and the upGPR measurements presented by Schmid et al. (2014) compared to the laser snow-depth sensor measurements. The RMSE was calculated over the dry-snow period only (4 January to 23 March 2011 and 3 December 2011 to 26 April 2012) assuming a constant wave speed *v* = 0.23 m/ns. For 2011–2012, results for the lower and upper antennas position are given.

Winter season	upFMCW		upGPR
	fullyAuto	semiAuto	semiAuto
	(cm)	(cm)	(cm)
2010–11	12	6	4
2011–12 (lower)	12	8	9
2011–12 (upper)	13	8	

## Appendix A FMCW link budget

For an estimate of the approximated link budget for the snow-to-air reflection we assumed an ideal homogenous snowpack with an ideal smooth surface. We used the permittivities of the dry-snow densities given in Section 2.1 and calculated the reflection coefficients *r*_*i*_ for the snow-to-air interface by Eq. (1). Their corresponding return losses

(A.1) $$R_{i}^{\left[\mathrm{dB}\right]}=20\;\log\left(r_{i}\right),$$

resulted in − 27 dB and − 15 dB for snow densities of 100 kg/m^3^ and 500 kg/m^3^, respectively. The received power and the total transmission loss were estimated by the radar equation

(A.2) $$P_{{\mathrm{RX}}_{i}}=P_{\mathrm{TX}}G^{2}\frac{\lambda^{2}|r_{i}|{}^{2}}{{\left(4\pi \right)}^{3}D_{i}^{4}}\sigma$$

with antenna gains *G*, reflection distance *D*_*i*_, wave length *λ* and radar cross section *σ*.

For estimating the reflected power of a single beat frequency (i.e. single power peak shape over 20 consecutive frequency bins in the power spectrum for *m* = 20), we calculated the radar cross section

(A.3) $$\sigma \approx 2\pi D_{i}^{2}\left(1-\cos\left(\frac{2\pi D_{i}^{2}\mathrm{ta}n^{2}\left(\alpha \left(D_{i}\right)\right)}{\lambda D_{i}}\right)\right)$$

of a circular reflection plane (e.g. Pouliguen et al., 2008) so, that the maximum run length difference of a spherical electromagnetic wave, reflected from the center of the plane (with incident angle = 0°) and that reflected from the border (incident angle *α*(*D*_*i*_)), was not larger than half of the physical resolution given in Eq. (4). From

(A.4) $$\frac{D_{\mathrm{res}}}{2}=2\left(\sqrt{D_{i}^{2}+D_{i}^{2}\mathrm{ta}n^{2}\left(\alpha \left(D_{i}\right)\right)}-D_{i}\right)$$

we obtained

(A.5) $$\mathrm{ta}n^{2}\left(\alpha \left(D_{i}\right)\right)=\frac{D_{\mathrm{res}}^{2}}{16D_{i}^{2}}+\frac{D_{\mathrm{res}}}{2D_{i}}.$$

By substituting Eq. (A.5) in Eq. (A.3) the resulting cross section was 10.9 m^2^ for a mean transmitting frequency of 1.5 GHz and a reflection distance of 1 m. The total estimated transmission losses (free space + reflection + cross section) were between − 65.3 dB and − 54.8 dB. With a transmitting power of + 20 dBm and antenna gains *G* of 11 dB, the estimated receiving power was in the range of − 23.3 dBm to − 12.8 dBm. By doubling the reflection distance this values decrease by 6 dB. However, we would like to point out that the above presented link budget does not take into account physical changes at the ideal snow–air interface during heavy snowfall (i.e. when the effective permittivity of air increases) and when multiple layers of varying density are present just below the snow surface.
